# Protein arginine methyltransferase 5 as a novel therapeutic target in solid tumors

**DOI:** 10.1016/j.gendis.2025.101796

**Published:** 2025-08-06

**Authors:** Ping Song, Fan Yang

**Affiliations:** a Department of Gastroenterology, Affiliated Hangzhou First People's Hospital, School of Medicine, Westlake University, Hangzhou, Zhejiang 310006, China; b Key Laboratory of Integrated Traditional Chinese and Western Medicine for Biliary and Pancreatic Diseases of Zhejiang Province, Hangzhou, Zhejiang 310006, China; c Hangzhou Institute of Digestive Diseases, Hangzhou, Zhejiang 310006, China; d Department of Vascular Surgery, Affiliated Hangzhou First People's Hospital, School of Medicine, Westlake University, Hangzhou, Zhejiang 310006, China; e Key Laboratory of Integrated Oncology and Intelligent Medicine of Zhejiang Province, Hangzhou, Zhejiang 310006, China

**Keywords:** Epigenetics, PRMT5, PRMT5 inhibitor, Solid tumor, Tumor immunology

## Abstract

Protein arginine methyltransferase 5 (PRMT5) is the primary type II methyltransferase that mainly catalyzes symmetric demethylation of arginine residues in both histone and nonhistone proteins. Increasing evidence has demonstrated that PRMT5 is indispensable in tumorigenesis and acquired therapeutic resistance in multiple malignancies. This review summarizes the clinical significance of PRMT5 in solid tumors such as lung cancer, breast cancer, and glioblastoma, its role in tumor immunology, and current clinical trials of PRMT5 inhibitors, and discusses the clinical status, current dilemma, and future perspectives of PRMT5 inhibition as a novel therapeutic strategy.

## Introduction

Epigenetics represents heritable but reversible changes in histone or DNA modifications.^1^ Epigenetic alterations consist of DNA methylation, histone protein modification, noncoding RNA regulation, and chromatin remodeling.^2^ Protein arginine methylation is one of the most common posttranslational modifications in mammals and is crucial in a breadth of cellular processes,^3^^,^^4^ which is mainly mediated by the protein arginine methyltransferase (PRMT) family.^5^ PRMTs work as writers to catalyze the transfer of methyl groups from S-adenosylmethionine (SAM) to arginine residues on both histone and nonhistone proteins, leading to modifications to protein–protein/RNA interactions, gene expression, and protein translation.^6^^,^^7^ PRMTs are classified into three types: i) type I PRMTs (PRMT1, 2, 3, 4, 6, and 8) catalyzes both asymmetrical dimethylation and monomethylation of arginine; ii) type II PRMTs (PRMT5 and 9) mediates symmetrical dimethylation and monomethylation of arginine; and iii) type III PRMTs (PRMT7) exclusively mediates monomethylation of arginine.^8^

PRMT5 plays an important role in a wide range of cellular processes and directly affects the proliferation and differentiation of tumor cells,^9^ including DNA repair,^10^^,^^11^ cell cycle,^12^ cell stemness and survival,^13^^,^^14^ primordial germ cell induction,^15^ ribosome biogenesis,^16^ RNA transport and Golgi body integrity,^17^ regulation of metabolism,^18^^,^^19^ hematopoiesis,^20^ and protein methylation and regulation of immunity.^21^, ^22^, ^23^ PRMT5 modulates gene expression in various ways, including transcriptional repression via histone methylation, modification of protein interactions, utilization of alternative gene promoters,^24^ and regulation of spliceosome fidelity and activity.^25^ In addition, PRMT5 can regulate posttranslational modifications and affect protein stability and signal transduction.^26^^,^^27^ The interaction between PRMT5 and other genes is listed in Table 1.

**Table 1:** Summary of interactions between PRMT5 and other key genes.

Gene	Targets/biological process	Oncogenic/TS/ND	Cancer type/Diseases	Reference
TP53	Cell growth, cell cycle, and apoptotic cell death	TS	Lymphoma, melanoma	83,142,143
MUSASHI2 (MSI2)	Cell growth, cell cycle	Oncogenic	Lymphoma	142
ST7	Cell-cycle, apoptosis, and cell migratory activity	Oncogenic	Glioblastoma	56
E2F1	Alternative RNA splicing, cell migration/invasion/adherence, protein half-life	Oncogenic	Myeloproliferative neoplasm, neuroblastoma, colon cancer	12,136,144,145
P65	Cell growth/migratory/colony-forming ability, DNA binding	Oncogenic	Colorectal cancer	75,146
KLF4	Protein half-life	Oncogenic	Breast cancer, neointimal formation	13,147
FOXP3	Regulatory T cell function	Oncogenic	Regulatory T cell	74
SmB, SMD1, SMD3	Cell viability, cell growth	Oncogenic	Lymphoma, prostate cancer	148,149–
SRSF1	Alternative splicing	Oncogenic	Acute myeloid leukemia	105
ZNF326	Alternative splicing, mRNA stability, DNA replication stress, interferon-stimulated genes	Oncogenic	Breast cancer, pan-cancer	71,150
EGFR	SHP1 recruitment, cell proliferation/migration/invasion	TS	Pan-cancer	151
Akt	Epithelial to mesenchymal transition	Oncogenic	Colorectal cancer	78
PDGFR	Protein degradation	ND		152
RUVBL1	Homologous recombination	ND		111
TDP1	DNA damage, protein degradation	ND		153,154
BCL-2	Nuclear translation of FOXO1 and its transcriptional activity	Oncogenic	Lymphoma	155
RAD9	Cell cycle checkpoints control	ND		156
PDCD4	Tumor growth	Oncogenic	Breast cancer	63
IFI16	cGAS/STING pathway activation	Oncogenic	Melanoma	22
TRIM21	DNA damage	Oncogenic	Osteosarcoma	157
hnRNPA1	Alternative splicing	Oncogenic	Pan-cancer	70
HNRNPH1	Alternative splicing	Oncogenic	Hepatocellular carcinoma	158
YBX1	NF-κB activation and its downstream target gene expression	Oncogenic	Colorectal cancer	159
Prdm1	T Cell differentiation	Oncogenic	Pan-cancer	51
Srebp1a	Metabolic reprogramming, lipogenesis	Oncogenic	Pan-cancer	18
WDR77	mRNA translation, cell growth	Oncogenic		160

Note: TS, tumor suppressive; ND, not determined; snRNP, small nuclear ribonucleoprotein.

The possible role and importance of PRMT5 in cancer have been preliminarily summarized.^28^, ^29^, ^30^ However, considering there is still a growing body of research showing that PRMT5 is deeply involved in the formation of treatment resistance to chemoradiotherapy and immunotherapy,^31^, ^32^, ^33^, ^34^, ^35^ a review of PRMT5 based on up-to-date literature is still necessary. In this review, besides the role of PRMT5 in solid tumors, we specifically focused on the clinical significance of the current application of PRMT5 inhibitors and the crucial role of PRMT5 in tumor immunology.^36^, ^37^, ^38^, ^39^

## The role of PRMT5 in lung cancer

Lung cancer remains the leading cause of cancer-related death worldwide.^40^ PRMT5 is overexpressed in lung cancer clinical specimens and cancer cell lines, and is positively correlated with tumor stage, lymphatic metastasis, and poor outcome.^41^ PRMT5 promotes the proliferation of lung cancer cells by diverse mechanisms, including interaction with Krüppel-like factor 5 (KLF5),^42^ activation of protein kinase β (Akt) signaling,^43^^,^^44^ and Smad-signal transducer and activator of transcription 3 (STAT3) activation.^45^ By regulating V-Erb-B avian erythroblastic leukemia viral oncogene homolog 3 (ErbB3), glioma pathogenesis-related 1 (GLIPR1) suppressed the proliferation of lung cancer cells and growth of xenografts. PRMT5 forms a stoichiometric complex with WD repeat domain 77 (WDR77), down-regulating the expression of GLIPR1 during lung tumorigenesis.^46^ PRMT5 also facilitates the metastasis of lung cancer.^47^^,^^48^ In addition, PRMT5 suppressed the transcription of miR-99 by symmetrical dimethylation of histone H4R3, leading to increased expression of epidermal growth factor receptor 3 (EGFR3) and activation of extracellular signal-regulated kinase 1/2 (Erk1/2) and Akt, thus promoting metastasis.^49^

Accumulating evidence indicated that PRMT5 played a vital role in tumor immunology. Increased expression of PRMT5 was associated with an immunosuppressive microenvironment.^50^ PRMT5 deficiency enforced Klrg1^+^ terminal CD8^+^ T cell development and eliminated anti-tumor activity.^51^ PRMT5 inhibition suppressed tumor viability as well as increased the tumor cell expression of cluster of differentiation 274 (CD274), thus activating the programmed cell death protein 1 (PD-1)/programmed cell death ligand 1 (PD-L1) axis and eliminating CD8^+^ T cell anti-tumor immunity. Combination of PRMT5 inhibition and anti-PD-L1 therapy led to increased and enhanced tumor-infiltrating cells.^52^

## The role of PRMT5 in glioblastoma

Glioblastoma (GBM) accounts for 50% of glioma cases. GBM consists of undifferentiated cells (cell stem cells), which adapt GBM to treatment-induced stress and therapeutic resistance, and the majority of mature differentiated cells.^53^ PRMT5 is aberrantly overexpressed in GBM, maintaining the self-renewal of cell stem cells and mature differentiated cells, facilitating the proliferation and migration of GBM cells, and promoting neurogenesis.^54^^,^^55^ The nuclear expression of PRMT5 indicated poor prognosis in GBM patients.^56^ Methylthioadenosine phosphorylase (MTAP) deficit is observed in approximately 40% of all GBM cases, and MTAP deficit leads to the accumulation of methylthioadenosine. PRMT5 sustained the expression of RING finger protein 168 (RNF168), up-regulated the expression of the E3 ubiquitin ligase Smad ubiquitination regulatory factor-2 (SMURF2), and destabilized H2AX (a histone variant that replaces conventional H2A in a subset of nucleosomes), leading to synergistically increased DNA impairment in MTAP-deficient GBM.^57^^,^^58^ Integrin β3 and osteopontin engagement promote the metabolic shift toward glycolysis and inhibition of mitochondrial oxidative phosphorylation in GBM cells, which was impeded by PRMT5 inhibition.^59^ LB100 is a protein phosphatase 2A (PP2A) inhibitor, but alone exhibited no survival benefit. Depletion of PRMT5 with LB100 treatment significantly reduced GBM tumor size and prolonged survival.^60^ PRMT5 depletion enhanced trametinib-induced cytotoxicity in patient-derived primary GBM neurospheres.^61^

## The role of PRMT5 in breast cancer

Breast cancer is the most common malignancy in women. PRMT5 is highly expressed in breast cancer and regulates the tumor suppressor programmed cell death 4 (PDCD4).^62^, ^63^, ^64^ PRMT5 promotes proliferation/migration/invasion, breast cancer stem cell renewal, and therapeutic resistance.^65^, ^66^, ^67^, ^68^, ^69^ The expression of PRMT5 is associated with high levels of heterogeneous nuclear ribonucleoprotein A1 (hnRNPA1) arginine methylation and aberrant alternative splicing.^70^ Accumulation of PRMT5/WDR77 catalyzes the methylation of the Zn-finger protein ZNF326, in turn leading to defects in alternative splicing and up-regulation of proliferation- and migration-related genes.^71^ PRMT5 also promotes WNT/β-catenin signaling through epigenetic silencing of pathway antagonists such as Dickkopf-related protein 1 (DKK1), leading to activated and enhanced expression of c-Myc, cyclin D1, and survivin (SVN) and subsequently enhanced proliferation of breast cancer cell lines.^72^ PRMT5 is recruited to the forkhead box P1 (FOXP1) promoter and facilitates symmetrically dimethylated histone H3R2 (H3R2me2s), SET1, and trimethylated histone H3K4 (H3K4me3) recruitment, enhancing breast cancer stem cell proliferation and self-renewal.^62^ Kruppel-like factor 5 (KLF5) is oncogenic and overexpressed in basal-like breast cancer. PRMT5 interacts with KLF5 and catalyzes the dimethylation of KLF5 at R57, which antagonizes Glycogen synthase kinase 3 beta (GSK3β)-mediated KLF5 phosphorylation and subsequent F-box/WD repeat-containing protein 7 (FBW7)-mediated ubiquitination and degradation, thus promoting breast cancer stem cell maintenance and proliferation.^65^ Transcription factor Slug forms a complex with PRMT5 and lysine-specific demethylase 1 (LSD1), exerting dual transcriptional activities on Slug to facilitate epithelial to mesenchymal transition.^73^ PRMT5 enhanced the nuclear translocation of RNA demethylase AlkB homolog 5 (ALKBH5), leading to the removal of N6-methyladenosine methylation of breast cancer susceptibility gene 1 (BRCA1) and enhanced DNA repair competency in breast cancer cells, which decreased doxorubicin efficacy.^67^

Immunotherapy emerged as a promising strategy for breast cancer. PRMT5 selectively methylates Kelch-like ECH-associated protein 1 (KEAP1) and thereby down-regulates the expression of nuclear factor erythroid 2-related factor 2 (NRF2) and its downstream target heme oxygenase 1 (HMOX1), facilitating anti-ferroptosis differentiation and sustaining proliferation and consequently acquired immunotherapy resistance in triple-negative breast cancer (TNBC).^68^ DS-437 disrupted PRMT5 function and impeded its complex with forkhead box P3 (FOXP3), reducing the regulatory T cells' function. Combination of DS-437 treatment enhanced the anti-tumor effects of anti-erbB2/neu monoclonal antibody by inducing tumor immunity.^74^ Based on conventional chemotherapy, endocrine therapy, immunotherapy, and other treatment modalities, the combination of PRMT5 inhibitors can be an effective strategy for breast cancer.

## The role of PRMT5 in colorectal cancer

Colorectal cancer (CRC) ranks as the third malignancy worldwide.^40^ PRMT5 is overexpressed in CRC,^70^ and its nuclear expression level is positively correlated with tumor stage and poor prognosis.^75^ PRMT5 promotes the degradation of methylated E2F-1 and inhibits DNA damage-induced apoptosis in CRC.^12^ By interaction with enhancer of zeste homolog 2 (EZH2), PRMT5 enhances EZH2 binding and trimethylated histone H3K27 (H3K27me3) accumulation and increases methylation levels of histone H4R3 and H3R8 on the cyclin-dependent kinase inhibitor 2B (CDKN2B) promoter, thereby promoting CRC proliferation.^76^ Perturbations in transforming growth factor-β (TGF-β) signaling play a crucial role in CRC tumorigenesis. PRMT5 catalyzes the methylation of SMAD4 at R361 to promote its nuclear translocation, inhibiting SMAD4-mediated dysregulation of the TGF-β signaling pathway and promoting TGF-β1-induced epithelial-to-mesenchymal transition and metastasis in CRC.^77^ PRMT5 also promotes epithelia-to-mesenchymal transition by activation of the EGFR/Akt/GSK3β signaling pathway.^78^ N-alpha-acetyltransferase 40 (NAA40) is highly expressed in CRC tissues compared with nonmalignant specimens. NAA40 promotes the transcription of PRMT5 and subsequently facilitates symmetrical dimethylation of histone H4R3 and thus promoting CRC growth. Loss of NAA40 suppresses PRMT5 transcription and inhibits tumor growth in the CRC patient-derived xenograft model, and inhibition of PRMT5 results in altered expression of key oncogenes and tumor suppressor genes, leading to suppressed CRC proliferation.^79^ In addition, PRMT5 functionally associates with PRMT6 and epigenetically represses the expression of CDKN2B and cyclin G1 (CCNG1), promoting CRC progression.^80^

## The role of PRMT5 in melanoma

The incidence of cutaneous melanoma has increased rapidly in recent years. The expression of PRMT5 is highly up-regulated in melanocytic nevi, melanoma, and metastatic melanoma.^81^ By interaction with SHANK-associated RH domain interactor (SHARPIN), PRMT5 regulates the transcription of SRY-box transcription factor 10 (SOX10) and melanocyte-inducing transcription factor (MITF) in a SHARPIN-dependent manner, inhibiting the transcriptional corepressor SKI and leading to melanomagenesis.^82^ Cyclin-dependent kinase 4/6 (CDK4/6) inhibitors, such as palbociclib, are currently in clinical development for melanoma. Acquired resistance to palbociclib is common and severely limits its therapeutic effect in melanoma. PRMT5 disrupts palbociclib-mediated splicing regulation of mouse double minute 4 (MDM4) precursor RNA, keeping p53 in a deactivated state and therapeutic resistance. PRMT5 inhibition enhances the splicing regulation of MDM4 precursor RNA and sequentially activates p53 and p21, inhibiting CDK2 activity and sensitizing the therapeutic response to palbociclib.^83^

PRMT5 plays a crucial role in regulating the tumor immunology of melanoma. Patients with advanced melanoma, one of the most immunogenic tumors, exhibit objective response rates to immune checkpoint therapy (ipilimumab, nivolumab, or pembrolizumab), ranging from 10% to 40%. Immune escape develops through various inhibitory mechanisms and eventually forms immunotherapy resistance in melanoma patients.^84^^,^^85^ By methylating interferon gamma inducible protein 16 (IFI16), the key molecule of cGAS/STING signaling pathway, PRMT5 attenuates cytosolic DNA-induced interferon (IFN) and chemokine expression and inhibits the transcription of NLR family CARD domain containing 5 (NLRC5), suppressing major histocompatibility complex class I (MHCI) antigen presentation. Combination of pharmacological (GSK3326595) or genetic inhibition of PRMT5 augmented secretion of IFN and chemokine production and increased MHCI abundance in melanoma, exhibiting a synergistic effect with immunotherapy against melanoma.^22^

## The role of PRMT5 in esophageal squamous cell carcinoma

Esophageal squamous cell carcinoma (ESCC) ranks as the eighth most common cancer worldwide. PRMT5 was up-regulated in ESCC clinical tissues as well as ESCC cell lines, enhancing the tumor characteristics of ESCC. PRMT5 knockdown increased the liver kinase B1 (LKB1) and the phosphorylated AMP-activated protein kinase (p-AMPK), and decreased the phosphorylated mammalian target of rapamycin (p-mTOR) expression, conferring anti-tumor effects in ESCC.^86^

Radioresistance is the main reason for the failure of radiotherapy in ESCC. By activation of PRMT5, stanniocalcin 2 (STC2) led to increased symmetrical dimethylation of histone H4R3 (H4R3me2s) and promoted DNA damage response through homologous recombination and non-homologous end joining pathways. In addition, STC2 participated in solute carrier family 7 member 11 (SLC7A11)-mediated ferroptosis in a PRMT5-dependent manner. Combination of PRMT5 inhibition could enhance the effect of STC2 inhibition to overcome ESCC radioresistance.^87^

## The role of PRMT5 in prostate cancer

Prostate cancer is the most frequently diagnosed malignancy in men. The PRMT5 expression in prostate cancer is significantly higher than that in benign tissue. Up-regulation of PRMT5 is associated with poor progression-free survival in prostate cancer. By inhibiting the transcription of calcium/calmodulin-dependent protein kinase II inhibitor 1 (CAMK2N1), PRMT5 enhanced the progression of prostate cancer.^88^ PRMT5 promotes prostate cancer cell proliferation by activating the transcription of the androgen receptor (AR).^89^ In addition, by recruiting the PRMT5 adaptor protein plCln to the AR promoter, PRMT5 functions as an epigenetic activator of AR transcription in castration-resistant prostate cancer.^90^

A combination of PRMT5 inhibition represents an important supplement for prostate cancer treatment. As inhibition of AR transcriptional activity or androgen synthesis remains the major mechanism for most available anti-androgen agents, PRMT5 knockdown completely suppressed the growth of xenografts in AR-positive LNCaP cells.^89^ Neuroendocrine differentiation promotes the development of treatment-induced neuroendocrine prostate cancer. Targeting PRMT5 prevented fractionated ionizing radiation-induced neuroendocrine differentiation and formation of neuroendocrine prostate cancer, sensitizing radiotherapy for prostate cancer.^91^

## The role of PRMT5 in cervical cancer

Cervical cancer is the most common malignancy threatening women's lives, especially in developing countries. High expression of PRMT5 was associated with poor prognosis.^92^ PRMT5 is physically associated with the transcription factor Snail and the nucleosome remodeling and histone deacetylase (NuRD) complex (metastasis-associated antigen 1/MTA1 is a core component of the complex) to form the transcriptional–repressive complex and promote the invasion and metastasis of cervical cancer. PRMT5 inhibitor EPZ015666 suppressed the epithelial-to-mesenchymal transition of cervical cancer.^93^ PRMT5 inhibition renders enhanced anti-tumor immunology and can be a potential target for cervical cancer immunotherapy. PRMT5 depletion in tumors resulted in increased and enhanced tumor-infiltrating T cells, promoting the expression of IFN-γ, tumor necrosis factor alpha (TNF-α), and granzyme B in T cells.^92^

## The role of PRMT5 in pancreatic cancer

Pancreatic cancer is a lethal malignancy worldwide, with its annual morbidity increasing.^40^ Systematic chemotherapy is still the first-line treatment for most pancreatic cancer patients, but with frustratingly limited therapeutic response and common treatment resistance.^94^^,^^95^ PRMT5 is significantly and aberrantly elevated in pancreatic cancer, and high expression of PRMT5 predicts poor prognosis.^96^ By activation of the EGFR/Akt/β-catenin signaling axis, PRMT5 can induce the expression of mesenchymal markers and suppress the expression of the epithelial marker, promoting epithelial-to-mesenchymal transition and subsequent metastasis.^97^ Metabolic reprogramming is one of the key hallmarks of cancer, and aerobic glycolysis is the best-characterized type, which supports cell proliferation under hypoxic conditions.^98^, ^99^, ^100^ PRMT5 catalyzes the methylation of the FBW7 promoter and suppresses its expression to promote aerobic glycolysis *in vitro* and *in vivo*, enhancing c-Myc stability and inhibiting its degradation, thereby promoting the progression of pancreatic cancer.^101^ The Hippo signaling axis is a well-known tumor suppressor pathway. PRMT5 symmetrically dimethylated the Hippo pathway initiator serine/threonine kinase 3 (STK3, also known as MST2) and suppressed MST2 autophosphorylation and kinase activity, thereby inactivating the Hippo signaling pathway in pancreatic cancer.^102^

PRMT5 inhibition represents a potential treatment choice for pancreatic cancer. Aberrant splicing is an important mechanism in pancreatic cancer tumorigenesis.^103^^,^^104^ PRMT5 inhibition led to perturbation in alternative splicing and diminished therapy resistance.^105^^,^^106^ Gemcitabine is a first-/second-line chemotherapy drug for pancreatic cancer, to which patients easily develop resistance with prolonged treatment cycles. PRMT5 knockout induces the accumulation of excessive DNA damage and impaired homology-directed DNA repair activity, providing synergistic vulnerability to gemcitabine.^96^^,^^107^

Pancreatic cancer is a “cold” tumor embedded by stroma, exhibiting an immunosuppressive microenvironment. Current clinical trials focusing on immunotherapy in pancreatic cancer have almost failed except for the COMBAT trial, which suggests that combining a C-X-C motif chemokine receptor 4 (CXCR4) antagonist may sensitize pancreatic cancer to immunotherapy.^108^, ^109^, ^110^ Increasing evidence suggests that PRMT5 plays a key role in tumor immunology. PRMT5 inhibition resulted in alternative splicing of histone acetyltransferase TIP60 and decreased methylation of the TIP60 cofactor RuvB-like protein 1 (RUVBL1), reducing FOXP3 acetylation regulated by TIP60, further resulting in an increase in degradation of FOXP3^+^ regulatory T cells and CD8^+^ T-cell influx.^51^^,^^74^^,^^111^

## The value of PRMT5 inhibitors in solid tumors

Increasing evidence has demonstrated that PRMT5 inhibition alone or in combination with other drugs exhibits promising anti-tumor efficacy in multiple cancers like MTAP-deleted tumors.^57^ Based on these findings, the translational value of PRMT5 inhibitors is under clinical trial evaluations. In this section, we summarize reported PRMT5 inhibitors in solid tumors based on the most up-to-date literature.

## Lung cancer

PRMT5 inhibitors exhibited anti-tumor activity for lung cancer. For example, SAM-competitive PRMT5 inhibitor PF-06939999 exhibited anti-tumor activity in splicing dysregulated non-small cell lung cancers with decreased liability of drug resistance.^112^ A novel small-molecule PRMT5 inhibitor, JNJ-64619178, suppressed proliferation in cancer cell lines, including lung cancer.^113^ MRTX1719 selectively inhibited PRMT5 and represents a promising therapy for about 10% of MTAP-deleted cancers, including non-small cell lung cancer.^114^ In addition, the combination of PRMT5 inhibitor EPZ015666 impeded the PRMT5-catalyzed methylation of MAX interactor 1 (Mxi1), impaired DNA damage repair, and enhanced radiotherapy-sensitivity in lung cancer.^115^

## Glioblastoma

PRMT5 inhibition leads to perturbation of RNA splicing, disrupting the proliferation and self-renewal of GBM cell stem cells.^54^ For MTAP-deficient GBM, EPZ015666 could suppress the activation of RNF168, up-regulate the expression of the E3 ubiquitin ligase SMURF2, and destabilize H2AX, leading to synergistically increased DNA impairment.^58^ Combination of PRMT5 inhibitor JNJ-64619178 enhanced trametinib-induced cytotoxicity in patient-derived primary GBM neurospheres.^61^ Small-molecular inhibitors targeting PRMT5 represent a promising strategy in GBM, and novel small-molecular inhibitors are under development to facilitate the clinical translation of PRMT5 inhibition in GBM.^116^^,^^117^

## Breast cancer

PRMT5 inhibition can be a helpful adjunct for breast cancer.^118^ PRMT5 small-molecular inhibitor tadalafil can decrease N6-methyladenosine RNA methylation and impede PRMT5-ALKBH5 axis-mediated doxorubicin resistance.^67^^,^^119^ CMP5 inhibits PRMT5 activity and decreases its recruitment and histone protein methylation in the promoters of Dickkopf-1 (DKK1) and DKK3, inactivating WNT/β-catenin proliferative signaling.^120^ Pemrametostat and estrogen receptor (ER) degrader fulvestrant synergistically inhibit the growth of ER-positive/RB-deficient cells derived from patient-derived xenografts, conferring benefits for CDK4/6 inhibitor-resistant ER-positive/RB-deficient breast cancer.^121^

## Ovarian cancer

PRMT5 is highly expressed and correlated with poor survival in ovarian cancer. PRMT5 symmetrically demethylated alpha-enolase (ENO1) to promote active ENO1 dimer formations, which increased glycolysis flux and accelerated tumor growth.^122^ PRMT5 was identified as a combination partner to poly (ADP-ribose) polymerase (PARP) inhibitors.^123^ Addition of PRMT5 inhibitor GSK3326595 to PARP inhibitor niraparib resulted in increased anti-tumor activity *in vitro* and *in vivo*.^124^

## Colorectal cancer

PRMT5 inhibitor GSK591 significantly inhibited the proliferation of CRC cells and induced cell cycle arrest.^78^ Co-intervention of PRMT5 and EZH2 effectively suppresses CRC growth both *in vitro* and *in vivo*.^76^ Tadalafil impeded lactate dehydrogenase A (LDHA) expression and suppressed glycolysis, sensitizing CRC to 5-fluorouracil.^125^ PRMT5 knockout significantly increased expression of interleukin-12 alpha (IL12α), C–C motif chemokine ligand 22 (CCL22), and immunoglobulin heavy constant gamma 1 (Ighg1) in tumor-infiltrating B cells, leading to an increase in recruited T cells to the tumor and enhanced immunocytotoxicity.^126^

## Pancreatic cancer

Targeting PRMT5 alone or in combination with conventional therapy is an important new treatment strategy for pancreatic cancer. AlphaLISA-based high-throughput screening confirmed that candesartan cilexetil and cloperastine hydrochloride significantly inhibited PRMT5 activity and exerted anti-tumor effects.^127^ Application of PRMT5 inhibitor T1-44 enhanced the efficacy of TGF-β1 signaling pathway inhibitor vactosertib, leading to prolonged survival of tumor-bearing mice. Delivery of the PRMT5 inhibitor EPZ015666 with exosomes effectively inhibits the proliferation of pancreatic cancer cells.^128^ Intriguingly, patient-derived xenograft-based experiments revealed a highly PRMT5 inhibitor-sensitive subtype, for which application of PRMT5 inhibitors like GSK591 can induce DNA damage, G2/M cell cycle arrest, and apoptosis.^129^

## Current limitations and future perspectives of PRMT5 inhibition

PRMT5 participates in a wide range of cellular signaling pathways, genes, and protein expression, making it an attractive target. However, this also limits its clinical translation.^130^ PRMT5-mediated regulation of gene expression may exhibit synergy or antagonism with other methyltransferases. For example, PRMT7 catalyzes the methylation of distal H4R17 arginine residues on the histone tail and can enhance the efficiency of PRMT5-mediated methylation on histone H4R3 by positive cooperativity.^131^ The loss of asymmetric arginine methyltransferase PRMT1 sensitizes PRMT5 inhibition, showing a synergistic effect when combined with PRMT1 inhibitors in small cell lung cancer and pancreatic cancer cell models.^132^ CASP8 and FADD-like apoptosis regulator (CFLARL) is a critical anti-apoptosis protein that inhibits the activation of caspase 8 in mammalian cells. PRMT5 reduces the ubiquitination of CFLARL and increases its protein expression, while PRMT1 increases its ubiquitination and decreases its protein expression.^133^ Moreover, PRMT5 activates nuclear factor kappa B (NF-κB) signaling by symmetrical dimethylation of p65 at the R30 subunit, while PRMT1 restricts TNFα-induced NF-κB activation via asymmetric methylation of R30.^134^ Furthermore, PRMT5 has different regulatory effects on E2F-1 compared with PRMT1.^135^, ^136^, ^137^ PRMT5 and microRNAs (miRNAs)/long noncoding RNAs (lncRNAs) mutually regulate each other, although these have not been fully studied. PRMT5 functions as an epigenetic repressor of miRNAs to enhance c-Myc and cyclin D1 signaling, while it can also be regulated by miRNAs and lncRNAs.^138^^,^^139^ Current trials of PRMT5 inhibitors are listed in Table 2.

**Table 2:** Current clinical trials of PRMT5 inhibitors.

Drug	Conditions	Primary outcomes
GSK3326595	Early-stage breast cancer	The proportion of patients who achieve a complete cell cycle arrest, defined as a reduction in the proportion of Ki67 positively staining cells to ≤2.7%
GSK3326595	Myelodysplastic Syndrome (MDS) and acute myeloid leukemia (AML)	Percentage of participants with clinical benefit rate (CBR). Number of participants with non-serious treatment-emergent adverse events. Number of participants with AEs by Severity. Number of participants with dose limiting toxicities (DLTs). Number of participants with AEs leading to dose interruptions, dose reductions and treatment discontinuation due to AEs
GSK3326595	Solid tumors and Non-Hodgkin's lymphoma (Meteor 1)	Number of participants with any adverse events (AEs) and Serious adverse events (SAEs) Solid tumor cohorts: Overall response rate (ORR) Number of participants with any adverse events (AEs) and Serious adverse events (SAEs)
PRT543	Relapsed/refractory advanced solid tumors, relapsed/refractory diffuse large B-cell lymphoma, relapsed/refractory myelodysplasia, relapsed/refractory myelofibrosis, adenoid cystic carcinoma, relapsed/refractory mantle cell lymphoma, relapsed/refractory acute myeloid leukemia, and refractory chronic myelomonocytic leukemia	To describe DLTs of PRT543 To determine MTD To determine RP2D and schedule of PRT543
PRT811	Advanced solid tumor, recurrent glioma	To describe DLTs of PRT811 To determine MTD To determine RP2D and schedule of PRT811
JNJ-64619178	Neoplasms, solid tumor, adult non-Hodgkin lymphoma, and myelodysplastic syndromes	Part 1/2: Number of participants with DLTs
TNG462	MTAP loss and RAS mutant metastatic pancreatic adenocarcinoma (PDAC) or locally advanced or metastatic non-small cell lung cancer (NSCLC)	Phase 1: Maximum tolerated dose Phase 1: Dosing schedule Phase 2: anti-neoplastic activity
TNG462	MTAP-deleted solid tumors	Maximum tolerated dose Combination anti-neoplastic activity
AMG 193; docetaxel; comparator AMG 193 test tablet	Advanced MTAP-null solid tumors	Part 1/2: Number of participants who experience a DLT Part 1/2: Number of participants who experience a treatment-emergent adverse event Part 3: Objective response rate
AMG193	Advanced thoracic tumors with homozygous MTAP-deletion	Number of participants experiencing dose limiting toxicities (DLT) Number of participants experiencing treatment emergent adverse events (TEAE) Number of participants experiencing Serious adverse events (SAE)
AMG193	Advanced gastrointestinal, biliary tract, or pancreatic cancers with homozygous methylthioadenosine phosphorylase (MTAP)-Deletion	Number of participants experiencing dose limiting toxicities (DLT) Number of participants experiencing treatment emergent adverse events (TEAE) Number of participants experiencing Serious adverse events (SAE)
AMG193	Advanced methylthioadenosine phosphorylase (MTAP)-Null solid tumors	Number of participants experiencing dose limiting toxicities (DLTs) Number of participants experiencing treatment-emergent adverse events (TEAEs) Number of participants experiencing Serious adverse events (SAEs) Objective response (OR) per modified response evaluation criteria in solid tumors (RECIST) 1.1
AMG193	MTAP-deleted advanced NSCLC	Objective response (OR) per RECIST 1.1 Objective response (OR) Measured by computed tomography (CT) or Magnetic resonance imaging (MRI) and assessed per response evaluation criteria in solid tumors v1.1 Number of participants experiencing treatment-emergent adverse events (TEAEs) Number of participants experiencing events of interest (EOIs) Maximum concentration (cmax) of AMG 193 Time to cmax (tmax) of AMG 193 Area under the concentration–time curve (AUC) of AMG 193
TNG908	Locally advanced solid tumor	Phase 1: To determine MTD and dosing schedule of TNG908 Phase 2: To assess anti-neoplastic activity of TNG908 in patients with MTAP-deleted advanced solid tumors by RECIST v1.1 or modified RANO criteria
SCR-6920 capsule	Solid tumor, non-Hodgkin lymphoma	DLT, overall response rate
MRTX1719	Advanced solid tumors with homozygous methylthioadenosine phosphorylase deletion	Maximum observed concentration (cmax) Time of maximum observed drug concentration (tmax) Area under the concentration–time curve from time zero to the time of the last quantifiable concentration (AUC(0-T)) Area under the concentration–time curve from time zero extrapolated to infinite time (AUC(INF)) Terminal elimination half-life (T-HALF) Apparent total body clearance (CLT/F) Apparent volume of distribution during the terminal phase (Vz/F) Percentage of estimated part for the calculation of AUC(INF) (%AUC(INF)) Blood-to-plasma total radioactivity (TRA) ratio Total amount of administered dose recovered in urine (UR) Percent of administered dose recovered in urine (%UR) Renal clearance (CLR) in urine Total radioactivity in UR Total radioactivity in %UR Total radioactivity in total amount of administered dose recovered in feces (FR) Total radioactivity in percent of administered dose recovered in feces (%FR) Total amount of radioactivity recovered (rtotal) Total percent of radioactivity recovered (%TOTAL) TRA amount recovered and fraction of the radioactive dose in vomit if applicable
MRTX1719	Mesothelioma, non-small cell lung cancer, malignant peripheral nerve sheath tumors, solid tumor, pancreatic adenocarcinoma, and advanced solid tumor	Phase 1: Number of patients who experience a DLT Phase 1/1B: Number of patients who experience a treatment-related adverse event Phase 2: Objective response rate Phase 2: Duration of response Phase 2: Progression-free survival Phase 2: Overall survival
IDE397; docetaxel; paclitaxel; gemcitabine; nab paclitaxel; pemetrexed	Solid tumor harboring MTAP deletion	DLTs of IDE397 DLTs of IDE397 in combination with either docetaxel or paclitaxel MTD and/or RP2D of IDE397 MTD and/or RP2D of IDE397 in combination with either docetaxel or paclitaxel To evaluate the preliminary anti-tumor activity of IDE397 in combination with expansion arms
AMG193 in combination with IDE397	Advanced methylthioadenosine phosphorylase (MTAP)-Null solid tumors	Number of participants experiencing dose limiting toxicities (DLTs) Number of participants experiencing treatment-emergent adverse events (TEAEs) Number of participants experiencing Serious adverse events (SAEs) Objective response (OR) per modified response evaluation criteria in solid tumors (RECIST) 1.1
PF-06939999 dose escalation; PF-06939999 monotherapy; PF-06939999 in combination with docetaxel	Advanced solid tumors, metastatic solid tumors	Number of participants with DLTs Number of participants with treatment-emergent adverse events Number of participants with laboratory abnormalities Objective response rate
AZD3470	MTAP-deficient advanced/metastatic solid tumors	Incidence of adverse events and serious adverse events Incidence of DLTs
AZD 3470	Relapsed/refractory hematologic malignancies, non-hodgkin lymphoma, and hodgkin lymphoma	Incidence and severity of adverse events and serious adverse events Incidence of DLTs (dose escalation only)
PEP08		
BGB-58067	Advanced solid tumors	Number of participants with adverse events and Serious adverse events Maximum tolerated dose (MTD) or Maximum administered dose (MAD) Recommended Dose(s) for expansion (RDFE[s]) of BGB-58067 Recommended phase 2 dose (RP2D) of BGB-58067 Objective response rate (ORR)
BMS-986504	Recurrent GBM	BMS-986504 concentration in tumor tissue Number of treatment-emergent adverse events Number of treatment-related adverse events Number of Serious adverse events Number of clinical laboratory abnormalities Number of drug-related toxicities
BAY 3713372	MTAP-deleted solid tumors	Number of participants with treatment-emergent adverse events (TEAEs) Number of participants with treatment-emergent serious adverse events (TESAEs) Severity of treatment-emergent adverse events (TEAEs) and treatment-emergent serious adverse events (TESAEs) Incidence of dose-limiting toxicities (DLTs) Number of participants with DLTs Maximum concentration (cmax) of the respective dosing interval of BAY 3713372 Area under the curve (AUC) of the respective dosing interval of BAY 3713372
TNG456 alone and in combination with abemaciclib	Solid tumors with MTAP loss	Maximum tolerated dose Anti-neoplastic activity Single agent Anti-neoplastic activity combination treatment

Note: DLT, dose limiting toxicity; MTD, maximally tolerated dose; RP2D, recommended phase 2 dose;

To promote the clinical application of PRMT5 inhibitors, it is necessary to elucidate the regulatory mechanism of PRMT5 and find efficient biomarkers for assessing the effectiveness of PRMT5 inhibition. The current research on PRMT5's function and regulatory mechanisms mainly relies on gene knockdown/knockout technology and transcriptome analysis, which may not comprehensively and accurately reflect the diversity of PRMT5's functions and regulatory mechanisms. Excitingly, multiomics such as proteomics and spatial omics, CRISPR screening, organoids, and other advanced technologies are applied to establish a comprehensive understanding of the regulatory mechanisms of PRMT5, including its epigenetic regulation, transcriptional control, RNA transport, protein stability, and posttranslational modifications, as well as their relative importance and interdependencies.^140^^,^^141^

## Conclusion

PRMT5 exerts important epigenetic regulation on histone and nonhistone proteins. Targeting PRMT5 may be an effective therapeutic strategy for solid cancer. As targeted drugs against PRMT5 are under development and clinical trials evaluation, the underlying mechanisms will be further deciphered, and the specificity and effectiveness of these drugs will be improved.

## CRediT authorship contribution statement

**Ping Song:** Writing – review & editing, Resources, Writing – original draft. **Fan Yang:** Resources, Writing – original draft, Writing – review & editing.

## Funding

This work was supported by the Zhejiang Provincial Foundation of Natural Science (China) (No. LQ23H160046), the National Natural Science Foundation of China (No. 82202876), and the Medical and Health Research Project of Zhejiang Province, China (No. 2023RC225).

## Conflict of interests

The authors declared no conflict of interests.
